# Mindfulness-Based Cognitive Therapy (MBCT) for Health Anxiety (Hypochondriasis): Rationale, Implementation and Case Illustration

**DOI:** 10.1007/s12671-013-0271-1

**Published:** 2014-01-21

**Authors:** Christina Surawy, Freda McManus, Kate Muse, J. Mark G. Williams

**Affiliations:** 1 Department of Psychiatry, Warneford Hospital, University of Oxford, Oxford, OX37JX UK; 2 Department of Psychiatry and Oxford Cognitive Therapy Centre, Warneford Hospital, University of Oxford, Oxford, OX37JX UK

**Keywords:** MBCT, Mindfulness, Health anxiety, Hypochondriasis

## Abstract

Recent research has shown that mindfulness-based cognitive therapy (MBCT) could be a useful alternative approach to the treatment of health anxiety and deserves further investigation. In this paper, we outline the rationale for using MBCT in the treatment of this condition, namely its hypothesised impact on the underlying mechanisms which maintain health anxiety, such as rumination and avoidance, hypervigilance to body sensations and misinterpretation of such sensations. We also describe some of the adaptations which were made to the MBCT protocol for recurrent depression in this trial and discuss the rationale for these adaptations. We use a case example from the trial to illustrate how MBCT was implemented and outline the experience of one of the participants who took part in an 8-week MBCT course. Finally, we detail some of the more general experiences of participants and discuss the advantages and possible limitations of this approach for this population, as well as considering what might be useful avenues to explore in future research.

## Introduction

Anxiety about health is a common and distressing problem, affecting most people at some point in their lives and becoming clinically significant for up to 5 % of the general population at any one time (Gureje et al. 1997). Although concerns about being or becoming ill are familiar to many, it is the escalation of transient worries to a chronic preoccupation with the fear of either having or developing a serious medical illness which characterises the diagnosis of severe health anxiety (hypochondriasis). Several psychological therapies have been shown to be helpful in treating health anxiety (see Thomson and Page (2007) for a review), with the strongest evidence being for cognitive–behavioural therapy ((CBT), e.g. Sorensen et al. 2011). However, some studies of CBT for health anxiety have reported that as few as 30 % of eligible participants agreed to participate (Barsky and Ahern 2004) and dropout rates as high as 25–30 % have been reported (Greeven et al. 2007), indicating that existing psychological interventions may not always be acceptable to patients with health anxiety. Taken together, these data suggest that there is a need for more treatment options for this condition.

Mindfulness-based cognitive therapy (MBCT) builds on the strength and success of CBT and has at its heart a similar model for understanding health anxiety, but offers the possibility of change in a rather different way, which might be more acceptable to some sufferers. Preliminary results of studies evaluating MBCT for health anxiety report encouraging results. An initial pilot study (*N* = 10) reported that MBCT produced significant improvements in health anxiety, disease-related thoughts and somatic symptoms, which were sustained at 3-month follow-up (Lovas and Barsky 2010). More recently, a randomised clinical trial comparing MBCT to usual services (*N* = 74) reported that those allocated to MBCT were less likely to meet criteria for the diagnosis both immediately following the intervention and at 1 year follow-up (McManus et al. 2012). In addition, both these studies and a qualitative study of MBCT for health anxiety (Williams et al. 2011) reported MBCT to be an acceptable and beneficial treatment to patients with health anxiety. In this paper, we outline the rationale for MBCT in the treatment of health anxiety, describe the clinical methods used and illustrate this with a case example from our recent trial (McManus et al. 2012).

Recent developments in psychological interventions have included MBCT (Segal et al. 2002). This class-based programme was originally designed to provide accessible relapse prevention for recurrent depression by targeting the cognitive processes that underlie vulnerability to relapse, such as rumination and high cognitive reactivity (Teasdale et al. 1995). It has been shown to significantly affect these processes (e.g. Hargus et al. 2010; Kuyken et al. 2010; Raes et al. 2009) and reduce the risk of relapse of depression (Piet and Hougaard 2011). In recent years, MBCT has been gaining momentum in the treatment of a broader range of mental health problems including anxiety (Hofmann et al. 2010; Orsillo and Roemer 2011)

MBCT combines the training of mindfulness, through meditation practices, with psycho-educational components drawn from CBT. Formal and informal meditation practices are taught so that participants can learn to cultivate direct experiential awareness and non-judgmental acceptance of whatever arises in each moment, including negative mood states and anxiety. In his paper on mindfulness and psychological processes, Williams (2010) describes how, in depression, low mood can trigger a host of mental simulations or narratives which are then treated as real threats and real losses by primitive neural pathways in the brain. The difficulty then arises when the mode of processing which is applied in order to work with these mental simulations or narratives is the ‘doing’ mode of mind. This is a mode of mind which essentially tries to ‘solve’ the emotional problem by bringing in memories about the past and images about the future and setting goals in order to help find a solution. However, while this ‘doing mode’ is a useful and vital strategy for many day to day tasks, such as getting from A to B, when applied to mental events, it serves only to increase levels of rumination or suppression and increases a sense of helplessness and distress, as well as reducing attentional capacity.

Traditionally, CBT has intervened at both the level of emotional expression and also at the conceptual level (Beck 1976), helping people to understand and reframe the narratives and mental models associated with the emotion. Mindfulness training aims to teach access to a ‘being mode’ of mind, i.e. to attend to the unfolding of experience moment by moment with openness and non-judgment. This enables people to see more clearly the mind’s tendency to elaborate and create narratives which are then taken to be reality and also the *reaction* to this tendency, i.e. to want positive states to carry on, negative states to end and neutral states to be more exciting.

## Rationale for MBCT in Health Anxiety

There are several reasons to hypothesise that MBCT may be helpful in the treatment of health anxiety. First, MBCT is directly concerned with developing a new and more accepting relationship to experience. Whilst in CBT, the many ways in which thoughts about illness are challenged may have a similar effect in terms of the patient eventually beginning to view their thoughts more objectively, this is the explicit aim in MBCT. Second, many of the mechanisms purported to be responsible for the maintenance of health anxiety in cognitive behavioural models (Salkovskis and Warwick 2001) are likely to be impacted by MBCT. These include worry, rumination and avoidance, the role of hypervigilance to body sensations and misinterpretation of such sensations and intolerance of uncertainty. We will discuss four ways in which MBCT is likely to have an effect on these mechanisms below.

### Responding Instead of Reacting

Cognitive–behavioural conceptualisations suggest that triggers to anxiety about health, such as noticing a bodily sensation and assigning a negative interpretation to it, are responded to by vacillating between suppression and avoidance and by ruminating on the possible meanings of the sensation (Salkovskis and Warwick 2001; Wells 1997). Such responses have the effect of increasing the preoccupation, thereby maintaining the anxiety. This is characteristic of the doing mode of mind, which aims to reduce unpleasant emotional states by trying to find solutions to the ‘problem’ of distress in ways which are not always helpful. So, for example, noticing a shortness of breath may trigger an image of the individual’s funeral (Muse et al. 2010). This increases anxiety and the sense of uncertainty about the future, which intensifies the shortness of breath as well as triggering other physiological responses of anxiety, increasing the body sensations. Strategies to try to solve the distress are then activated, such as trying to suppress or avoid the sensations, images and associated emotions or ruminating about potential explanations for the symptom. Rumination has been shown to maintain health anxiety (Marcus et al. 2008) and there is evidence that MBCT can reduce it (Heeren and Philippot 2011; Michalak et al. 2011).

MBCT does not aim to change thoughts and images, but to reduce their impact by encouraging a decentred approach to them and to the reactivity that arises in relation to them. The aim is to break the cycle of escalation that might otherwise lead to anxious preoccupation (with its associated behaviours such as checking or reassurance seeking), habitual avoidance or rumination. This capacity to process events with a different mode of mind (‘being’ rather than doing) introduces the possibility of making choices about how to *respond* in a flexible way rather than *react* in a habitual way. So, for example, a patient is encouraged to allow a distressing image to be present and see it for what it is (a distressing simulation of a possible event, rather than a representation of reality) and to observe that bodily sensations vary and do not necessarily require any immediate intervention. Of central importance in the MBCT approach to developing flexibility of responses to distressing experiences is the capacity to approach and engage with the experience, rather than to habitually attempt to move away from, or avoid it, simply because it is unpleasant.

### Exploration of Body Sensations

Cognitive–behavioural conceptualisations of health anxiety have also highlighted the role of hypervigilance to bodily sensations, and this is supported by experimental studies of attentional bias in health anxiety (Rassin et al. 2008). Furthermore, there is preliminary evidence that training in attentional control strategies can be beneficial to patients with health anxiety (Papageorgiou and Wells 1998). A central tenet of MBCT is changing the mode of mind within which a person views their experience from problem solving or doing mode to an attitude of acceptance and exploration of body sensation (being mode). A distinction is drawn between the direct experience of the raw sensations (e.g. a tingling sensation in the hand) and the meanings and mental constructions that may have become associated with them (e.g. ‘this means I have a serious illness’). So rather than re-focussing attention away from the body, the meditation practices within the MBCT programme guide participants in developing curiosity towards body sensations, registering how these feel and observing how their minds and bodies react to them. The attitudes of compassion, warmth and non-judgmental acceptance are explicitly and implicitly encouraged when attending in this way (Segal et al. 2002; Kuyken et al. 2010). The practices and the whole tone of the classes are intended to foster an attitude of kindly curiosity towards all experience which has the additional and important effect of not only allowing flexible responding in the face of anxiety, but fosters an overall attitude of kindness towards the self rather than harsh judgments and self-criticism, particularly in the face of setbacks. By increasing a compassionate stance, MBCT can potentially reduce experiential avoidance, relieve distress and promote well-being and resilience (Kuyken et al. 2010).

### Engagement with the Present Moment

Borkovec and Sharpless (2004) propose that anxiety has the effect of ensnaring people in a future-oriented world in which they are out of touch with present moment reality, instead living lives in which their bodies and minds are reacting to mental constructions of reality. Consequently, they experience ‘little joy and little contact with present moment information’ (Borkovec and Sharpless 2004, p. 209). Way et al. (2010) showed that being routinely out of touch with present moment experience was linked to chronic over-reactivity of the limbic system and to stress and emotional reactivity. One of the aims of MBCT is to recover the engagement with ordinary moments in life, which can not only be pleasurable in itself, but is also incompatible with being locked into self and future-oriented elaborations, which are typical in health anxiety (Muse et al. 2010).

### Providing Skills to Prevent Relapse

A final advantage of MBCT is its suitability to a chronic episodic condition, such as severe health anxiety. As health anxiety exists on a continuum and health concerns are something that will arise for everyone from time to time, an intervention that provides individuals with the skills for responding to these concerns over the longer term is likely to have advantages with respect to relapse prevention. MBCT aims to reduce the likelihood of relapse by teaching participants to notice their unique early warning signs and providing them with a set of skills which can be used to ‘nip in the bud’ an escalation of normal health concerns into an episode of severe health anxiety.

## Adaptations to the 8-Week MBCT Programme

Although the MBCT core values and principles remain constant whichever client group or problem focus is being worked with, as the MBCT course was originally devised for treating recurrence of depressive relapse (Segal et al. 2002), it is important to note that the differences in the nature of the problem—especially the factors that maintain the disorder—are different from depression and mean that some adaptation is required for health anxiety (see Teasdale et al. (2003), for discussion of matching MBCT to the formulation of the problem).

The programme begins with an individual ‘orientation session’ in which the MBCT teacher assesses a patient’s suitability for the programme, collaboratively develops an individual problem formulation, explores how MBCT might be helpful and discusses what to expect during the course.

The remaining sessions are delivered in a group format over eight weekly sessions of 2 h each, as outlined in Segal et al. (2002). As with MBCT for depression, participants are asked to make a commitment to the course in terms of daily practice of meditation practices and other homework exercises. The relationship with the participants is that of a teacher with a class, with the subjects of discussion typically being about the experiences observed by the participants during meditation and other exercises.

### Sessions 1–3

The first three sessions closely follow the structure outlined in the MBCT programme for depression (Segal et al. 2002). Participants are encouraged to attend to automatic responding and tune into how often in daily life they do things automatically without engagement. This is highlighted in the first exercise in which they are asked to bring their full attention to eating a single raisin and notice how this is different from how they may typically approach the same situation. Corresponding home practice of deliberately engaging in this way with routine activities such as eating, walking or showering is encouraged.

The body scan (Kabat-Zinn 1990) is used to encourage participants to become aware of, and experience fully, sensations in the body just as they are. Participants are encouraged to notice the reactions of the mind and also how quickly the mind shifts from one topic to another. Having noted the wandering mind, participants practice returning their attention gently but firmly to a present, single focus of the body and breath. The emphasis here is on learning to accept the mind’s wandering nature and to recognise that sensations may produce particular thoughts and images and emotional reactions, whilst also learning that it is possible to refocus attention.

Psycho-education elements are included, such as the recording of pleasant and unpleasant events, to demonstrate how many positive moments are missed when one is constantly absorbed in thinking, and how there can be a tendency to want these moments to go on, colouring the experience of the present. Similarly, recording of unpleasant moments helps to encourage people to bring their attention to the experience, regardless of its valence, and to reduce avoidance of unpleasant emotions. For both pleasant and unpleasant situations, patients are encouraged to record thoughts as if ‘verbatim’, together with associated emotional feelings and bodily sensations.

Sitting meditation is introduced to build on what is learnt from the body scan, teaching that focus on the breath can have a steadying and anchoring effect, and then widening awareness to include the possibility of a more detailed observation of sensations in the body including noticing bodily markers of reactivity. Participants are encouraged to use the ‘breathing space’, a short practice which encourages present moment awareness in everyday life, in as many situations as possible, encouraging greater decentring from thoughts and feelings and attentional stability, as well as greater engagement with moment-by-moment experience.

### Session 4

**Fig. 1:**
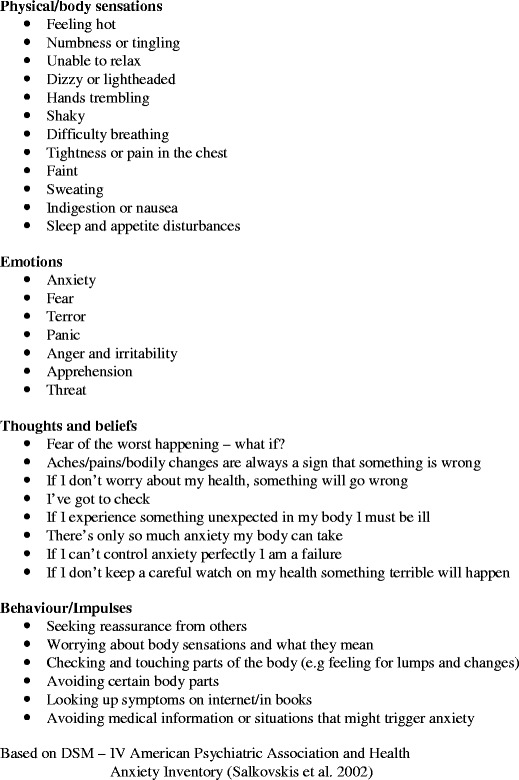
The territory of anxiety

The fourth session has a more educational focus. The original MBCT protocol for depression focuses on specific markers of depression, so this has been modified to focus instead on health anxiety. The triggers, thoughts, emotions and behaviours that are part of the territory of anxiety about health are drawn out of group discussion (see Fig. 1). Participants are encouraged to relate what has come up during their practice of mindfulness to the processes involved in maintaining health anxiety. The meditation practice helps them to see more clearly what is taking place in their experience, and this awareness can open up the possibility of choosing their responses rather than reacting habitually or automatically, for example by seeking reassurance or shutting off their feelings. The sitting meditation practice here also includes awareness of thinking and fostering a decentred attitude to thoughts and images, for example recognising the patterns that are common to many participants (future orientation, catastrophic themes). Participants are also encouraged to develop a curiosity about the physical markers of aversion, i.e. not wanting to have an experience, for example a lurch in the abdomen or spacing out.

### Sessions 5–8

**Fig. 2:**
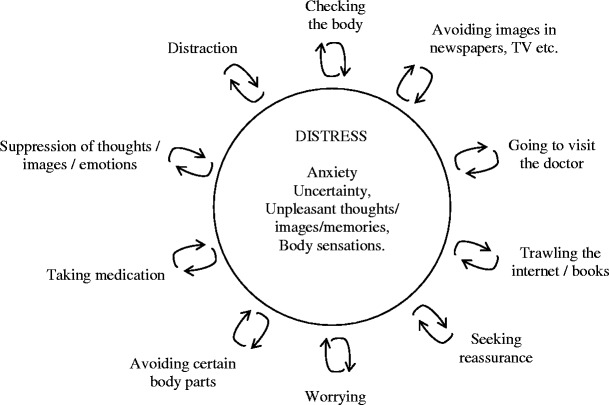
Identifying strategic maintenance processes in session 5. Adapted from Butler et al. (2008)

The remaining sessions focus on encouraging participants, through the meditation practices and educational components, to deliberately orient attention towards emotional and physical experiences that might once have elicited avoidance reactions. Encouraging patients to allow these to remain in awareness, they learn to acknowledge with kindness their usual patterns of reactivity. The format of these sessions is the same as in the original MBCT for recurrent depression protocol, but the educational components focus on the processes and content of the material central to maintaining health anxiety. For example, fear and uncertainty are the central emotional reactions rather than low mood and self-criticism. In session 5, the educational aspects of the class draw out how reactions to fear or discomfort, such as avoidance or trying to control the experience by analysing, worrying or seeking reassurance, may exacerbate anxiety and negative mood states and how mindfulness practices provide the opportunity to experiment with bringing a kindly awareness to the difficulties as experienced in bodily sensations and to experiment with alternative ways of responding (see Fig. 2). In session 6, thoughts and images typical of health anxiety are reflected on, as are ways in which the practice of meditation can enable participants to view these as ‘events in the mind’ which they can choose whether or not to engage with.

Attention is also given to participants’ broader lives. Reducing stress generally may have a positive impact on participants’ health worries, and in session 7, participants are encouraged to take a mindful stance on their current activities. As in MBCT for depression, they are asked to pay attention to how they are spending their time and what impact this has on them. Nourishing (pleasant or fulfilling) and depleting (draining or stressful) activities are monitored and participants are encouraged to reflect on the balance of activities in their life. For participants with health anxiety, this exercise can give them other options for action which are different to engaging in the usual reactions (e.g. trawling the internet) when anxiety arises, i.e. ‘what can I do to enhance my experience of being here right now, given that I am being pulled into an imagined and gripping future scenario in my mind?’ Towards the end of the course, as in the original protocol for depression, participants are encouraged to consider what might alert them to an episode of health anxiety and how they might respond as well as reflecting on how they might support themselves to continue with the practices.

In summary, the MBCT core values and principles have remained constant, but the nature of the problem influences how the approach is implemented. While many of the exercises in MBCT for health anxiety are modelled on those used in treating depressive relapse, the focus is on the particular issues and processes which are inherent in this problem and the rationale and educational aspects relate to the understanding of how health anxiety is maintained.

## Case Illustration

In order to bring to life what can be learned by engaging in MBCT as adapted for health anxiety, a case example is described below. Names and identifying details have been changed to preserve confidentiality.

### Background

Raymond was in his 40s, married and in full-time employment. He had experienced health anxiety and panic attacks since undergoing a serious heart operation some 12 years earlier. As regards his history, Raymond did not report any significant difficulties in his childhood but did describe his father as a ‘worrier’ and reported that his family or origin subscribed to the view that worry would prepare you for potential disasters of all kinds. Raymond had not received any psychological treatment previously, but had been taking antidepressant medication for several years. Raymond routinely monitored his breathing, and when he noticed any change (e.g. breathlessness), he became concerned that it meant that he was about to suffer a stroke or a brain haemorrhage if he was unable to get enough oxygen in. Over time his health concerns had generalised, and any unexpected bodily sensation, especially in his upper body (e.g. chest pain), would lead to the thought that there was something seriously wrong which needed medical attention. He experienced high levels of anxiety and worry from the moment he awoke and throughout the day and described himself as fed up and tearful most of the time. In addition, his relationship with his wife was suffering due to his low mood and irritability and the restrictions his anxiety placed on their lives (e.g. not travelling abroad because he did not trust the medical care outside the UK). Raymond frequently sought reassurance from his wife and his GP for his health concerns.

### Orientation Session: Providing a Rationale for MBCT

At the hour-long orientation session, the MBCT teacher explored with Raymond the ways in which MBCT might be helpful. The discussion centred on how the course might help him to recognise the way his mind was creating catastrophic scenarios based in the future and offer a way of relating to them differently, i.e. as passing thoughts, rather than in a reactive and habitual way. We also talked about how it was possible to understand that the mind creates these scenarios, given a history of life-threatening illness, but that the problem arises when the reaction to these mental events is based on the sense that they are real. We discussed how MBCT might help him to discover that there is a difference between physical sensations and the thoughts he was having about them and, in doing so, perhaps help him to develop a more positive and accepting relationship to his body. Raymond was only aware of intense sensations which were immediately linked to thinking and struggled to recognise that there was a difference between the thoughts and the raw sensations. His concerns were often associated with worry or impulsive behaviour (visiting the GP, shouting at his wife) and we talked about how MBCT might help him to respond rather than react: to choose different and more skilful responses rather than what came automatically to him. Since so much potential pleasure was missing from his life because of the almost constant worry, checking and reassurance seeking, the skills in awareness he would learn might help him to re-establish a sense of connection with his wider life and family.

### Early Sessions: Working with Sensations in the Body

One of Raymond’s concerns was that his health anxiety would be exacerbated through participating in the course. Specifically, he feared that focussing on his thoughts would be unhelpful and would increase his level of anxiety, which was an indication of how realistic he found them and how much of the time he spent pushing them away. Indeed he did find it a challenge to focus on the body as he rapidly became drawn into the thoughts that arose when he did so. In the first two sessions, Raymond was very tense. He sat hunched forward gripping the chair. He described the guidance in the body scan to focus attention on and explore physical sensations in the region of the heart as very frightening. In the same way, in the sitting practice, Raymond experienced focussing attention on the breath as very difficult and only managed it by reassuring himself that it was OK, saying to himself ‘it will be alright’. While he was in this verbal mode, he was clearly less in touch with the actual sensations. He described this experience of sitting with the breath later as being ‘almost intolerable’ in terms of the anxiety it provoked and that he desperately wanted to get up and leave. The MBCT teacher gently encouraged him to notice any thoughts that came up in reaction to the focus on the body and to see if it was possible to let them be, but not follow them, and to anchor his awareness to sensations by putting his hands first on the chair and then on his abdomen. Doing this, he firstly became aware of the direct sensations of contact with the chair and his hands (a part of the body which was not so threatening) and began to distinguish sensations in his hands from thoughts about his hands which he recognised as a different sort of phenomenon. Later on, Raymond talked about his experience of directing his attention towards the sensations he most feared (breath sensations, especially when they fluctuated), briefly allowing himself to experience the sensations of the breath by putting his hands on his abdomen, noticing how the thoughts developed in reaction and letting them be and coming back to the sensations in his abdomen. He realised that he had not been at all aware of sensory experience here, only of the thoughts about his rapid breathing. He described the insight that the sensations of the breath and the thoughts about them were different phenomena, as a ‘revelation’. He had also learnt that it was possible to slowly approach the sensations in the body, directing his attention towards them, as an alternative to getting caught in his thinking. Note that there was no attempt to change Raymond’s thinking or sensations.

Later on in the classes, Raymond described how he was frequently able to say to himself ‘it’s just a sensation’ when he became aware of pain or discomfort, and actually moving into the experience of it in his body, which he experienced as very different from being caught up in a conceptual framework. He described the effect of this as changing the whole ‘threshold of anxiety’ and feeling much more relaxed in general. By continuing to do the body scan and sitting practices, Raymond had the opportunity to practice observing his experience of sensations changing moment by moment and providing him ultimately with a focus for his attention which was steadying rather than a source of threat. This was very different to the usual experience he had been having of *thoughts about* sensations spiralling out of control very quickly.

### Later Sessions: Working with Health Anxious Thoughts

As the course progresses, there is an increasing emphasis on helping participants discover a more decentred and friendly relationship to thoughts and images. Raymond had felt completely ‘out of control’, and his desperate attempts to either find answers to his thoughts or get rid of them were creating a vicious circle that exacerbated this feeling. He described a shift as he began to realise that the thoughts that were coming into his head were ‘just thoughts’ and that he could accept them, let them come into his mind and ‘not get carried away’. He realised that although the thoughts were going around in his mind, creating a scenario of what would happen in the future, he was physically present in the ‘here and now’ rather than in that scenario (‘it just is what it is, and this is the here and now’). He also found this ability to create space for his thoughts opened up the possibility for the emergence of other perspectives, for example ‘I am going to die’ was no longer the only option, but was also accompanied by a sense that this might not happen so imminently. He also found the psycho-educational elements of the course reinforced his meditation experience. In session 2, the teacher takes the class through an exercise drawn from the CBT tradition in which participants are invited to reflect on their thoughts and feelings in response to a verbally presented scenario. Raymond found this was very helpful in fostering a more decentred approach to thinking. He described realising that for any given scenario there were ‘101 other possible interpretations’, a realisation which helped him to relate to his thoughts differently and feel more in control, rather than being ‘pulled about’ by them. For example, rather than buying into the thought ‘I am having a heart attack’, Raymond described doing a short meditation (3-min breathing space) when he noticed this thought and becoming aware of the emergence of a sense of greater acceptance reflected in thoughts such as ‘well, this is here now, I’ve had these (pains) before, it’s no big deal, I’ll just carry on’. By the end of the course, Raymond noticed that many of his anxious thoughts about his health had just disappeared and, although at times he would experience a rush of them, especially when he was physically ill, they would not escalate into a panic attack.

### Learning Kindness

At the start of the course, Raymond was engaging in a lot of judgment about being ‘stupid’ and ‘weak’ because he was reacting with anxiety to what he later perceived as such trivial triggers (though not of course at the time he was experiencing them). Throughout the course, the gentle encouragement of the MBCT teacher and the other participants to acknowledge this critical tone with kindness enabled Raymond to feel recognised and more accepting of his own difficulties. The environment of the class was very helpful in dissolving this self-criticism and harsh judgment, as these were common themes across participants and Raymond described the importance of realising that health anxiety was something others were experiencing in the same way as he was.

### Reflecting on the Impact of the Course

By the end of the classes, Raymond was able to report that, for the first time in years, he had begun to enjoy life. He felt lighter and much less irritable with his wife and was much less inclined to seek reassurance, describing his attitude as ‘just being able to get on with things’. He talked about feeling surprised that what used to worry him so much was much less concerning ‘That just being aware of something, feeling something, it’s not stopping me or getting in the way. I’m not sitting dwelling on whether I’m about to be really ill, which I would have before’. He described noticing that he wasn’t concerned about his wife going away and leaving him on his own and he was planning his first holiday abroad for years. Raymond also reported that his anxiety was ‘at a much lower level’. In other words, rather than being ‘up there so that the slightest thing takes me way off’ it was less easily triggered, even by recurrence of his health problems. In the feedback that all participants complete at the end of the course, Raymond reported that the period during which had had been attending the classes had been the ‘least anxious period for many years, previously a day or two at a time was all I could manage’, even though at the start of the course his anxiety had briefly escalated. Raymond described a recent incident while travelling home in which he felt unwell just before he boarded the train. Previously, he would have spun into a cycle of panic and called his wife to come and drive him home. This time he paused, focussed on his breath and stepped back from his overwhelming thoughts of impending disaster, just letting them come and go, with a greater sense of acceptance and calm, and then, the whole thing ‘just dissipated’ and he got on the train home. The shift in Raymond’s capacity to relate in a more considered way to his experience of anxiety is evident in this description and is quite different to the reactive pattern which had become familiar over the years. In general, this shift has had a big impact both on his life and on the life of his spouse. Raymond’s health anxiety reduced by a clinically significant amount (30 %), from 35 to 24 on the Short Form Health Anxiety Questionnaire (SHAI), which was maintained over 1 year.

## Discussion

### A Different Relationship with Experience and Its Impact

Raymond’s changed relationship to his thoughts and body sensations seems to reflect a gentler, more accepting and less reactive quality, and the positive consequences of this on his health anxiety and broader life were also evident in other participants. For example, one participant noticed that when the thought ‘this is cancer’ comes up, his response is to wait and see what happens rather than rushing straight to the GP (‘before I would be in a real mess about it’) and that these thoughts were ‘less intense and less frequent—I don’t really see the images of my funeral any more’. He also reported that the thoughts which prevented him from driving, going out and seeing his friends (‘I’ll die on the way’) were no longer interfering with his ability to do these activities. Another reported that he was able to ‘laugh at and be interested in/curious about health related thoughts’ and was able to cope more calmly with a particularly stressful occurrence during the course in which his partner was involved in a health scare. He described the course teaching him that there was another way to work with unwanted thoughts. One participant who experienced vivid images reported that her circulatory problems would have sent her into ‘horror scenarios’, but now she was able to say to herself ‘it’s aches and pains’ and found that her distressing images were ‘not interfering’ with her ability to get on with life. Finally, a student whose friend had died in a car crash was experiencing symptoms including being too frightened to sleep (in case she died in her sleep) and gastrointestinal upsets which she automatically attributed to having a tumour. She had given up sport, stopped reading the paper in case she came across information about anyone dying young, developed a fear of car accidents (seeing them or reading about them as well as having one), constantly asked her mother for reassurance and checked her body for any lumps or signs of possible cancer. At the end of the course, she described being much more aware of the direct experience of her body and this helping her to tune into physical sensations, rather than getting caught up in her thoughts or emotions. She described feeling much more connected with her body, picking up sensations which indicated she was rushing around, as well as anxiety. This recognition also enabled her to start to appreciate the present more ‘I feel more connected with my body instead of rushing—enjoying the moment more and relaxing into it’. She took up dancing, an activity which she had not done for a long time.

This capacity to relate differently to experience and its wider effects were also reflected in the themes which emerged in a qualitative study of MBCT for health anxiety carried out by Williams et al. (2011). Participants’ experiences could be grouped into the following: validation and normalisation of my experiences through MBCT; an awareness of my anxiety cycle enables me to break it; acceptance of my experiences; a different outlook on my life in general; and change large enough for significant others to notice. All of these effects can be seen in the case of Raymond.

### Difficulties and Challenges

In working with people suffering from heath anxiety, we note a number of challenges which may be different to those faced in working with recurrent depression. As described above, some participants may find it difficult to engage with practices such as the body scan that highlights bodily sensations they have previously been avoiding. There may also be fear of letting go of attempting to control one’s experience or giving up previous coping strategies that have enabled participants to get by thus far. However, as we have also seen, exploring these previously avoided experiences can be transformative by bringing online a different mode of mind (being mode) to the usual problem solving or doing mode of mind, which comes with an entourage of unhelpful narratives about the future and past and which tends to intensify emotional difficulties. Careful instruction about doing this work gradually, which are integral to the meditation guidance (e.g. ‘exploring as much as you feel able to’), and giving people ways to ground themselves in the physical world when they feel overwhelmed (particularly using the senses of touch and vision) are important. For example, as described above, Raymond was encouraged to experiment exploring direct physical sensation by touching the chair he was sitting on with his hands before moving to focus on sensations of breathing which usually gave rise to fear-related thoughts.

Given the difficulties reported in group CBT for health anxiety, we wondered whether there would be difficulties inherent in the group format (Wattar et al. 2005). A common theme is a sense of shame about suffering from health anxiety and the idiosyncratic concerns and associated behaviours. In addition, hearing about the experiences and concerns of others could trigger thoughts of illness and fuel feelings of fear. However, in the qualitative study described above (Williams et al. 2011), even though this was true for a very small minority of participants, most reported that they found the group a validating and normalising experience, from which they derived benefit. Also, the number of people who dropped out of treatment was very small (2 out of 36; McManus et al. 2012). This may be because MBCT is ‘class based’ rather than ‘group based’, meaning that the environment is much more focussed on learning skills rather than discussing individuals’ specific health anxiety concerns.

Anxiety is a condition which, because of its very nature, is characterised by a feeling of urgency and impatience. Hence, we were also aware that the meditation practices participants were encouraged to do at home could prove too long. Participants had different experiences of this. For some, the experience of boredom and impatience were ‘grist for the mill’, i.e. they were very useful states of mind to encounter in order to learn how to relate to them differently. For others, sometimes this was just too much and the intensity of the experience undermined the desire to practice (The struggle to find time to practice was also reported as a theme for participants by Williams et al. 2011). On the whole, most participants engaged in some home practice and we were able to encourage them to at least begin a practice. There is a balance to be struck between having the time during practices to experience all the difficulties of impatience, boredom and other challenging emotional states and finding the practice overwhelming. So, practising for a shorter time is preferable to not practising at all. In fact, 60 % of participants reported that they routinely practised at home throughout the 8-week course, and 75 % reported that they practised at home for at least 5 weeks of the 8-week course, though this data is based on retrospective self-report and may therefore not be reliable.

Throughout the course, we felt it was very important to acknowledge the participant’s courage in coming at all, particularly in the face of fear, and in persevering when difficulties arose. Indeed, it has been our experience that it is through persevering in the face of difficulties that the most useful insights are gained, and that it is the practices that are initially most difficult that ultimately prove most useful and become favoured practices. However, this was very difficult to do for some participants. Indeed, it is important to remember that at the end of 8 weeks, it may not be a skill that is accessible to all, and that further research is needed to clarify who is most likely to benefit from participating in MBCT.

It is also important to note that the adaptations to the MBCT programme did not include a broader range of interventions which are integral to other treatments such as CBT. For example, we could have given more attention to working more explicitly with patients to decrease safety behaviours or included exercises on working with uncertainty. In the programme as we have described it, these themes were addressed as they arose, but adapting the programme further to give them more emphasis could certainly be investigated in future work, though this would need to be carefully considered in terms of how such adaptations are integrated into the programme as a whole and its main aims and intentions.

## Conclusions

Both MBCT and CBT offer a helpful conceptual framework for understanding the maintenance of health anxiety and draw on this to help patients understand their distress. Unlike CBT, in MBCT there is little focus on addressing the content and meaning of thoughts, rather the emphasis is on changing the awareness of and relationship with thinking. Whilst without detailed studies, it is difficult to know what mechanisms are most pivotal in accounting for change, we can perhaps hypothesise that those particularly core to MBCT may have a part to play. The extended practice through meditation of developing a friendly awareness towards thoughts, emotions and body sensations may be important in facilitating disengagement from rumination or other unhelpful strategies, developing a new and less reactive relationship with the body, facilitating flexibility of attention and in general developing an aware mode of being, characterised by freedom and choice, in contrast to a mode dominated by habitual, over learned, automatic patterns of cognitive–affective processing. In support of this, the development of mindfulness was shown to mediate the changes in health anxiety observed in participants in the trial (McManus et al. 2012). While research exploring the use of mindfulness in the treatment of health anxiety is in its infancy, such initial findings are promising and provide the basis for further investigation. In particular, the authors of that paper suggest that ‘it will be a priority for future studies to compare the impact of MBCT with alternative interventions’. This will give us a clearer indication of how best to offer MBCT within a clinical setting, either as a standalone treatment for those who cannot access individual CBT (or a different psychotherapeutic approach) or do not wish to, or as an adjunct to CBT. Given the episodic nature of the condition, more research on its impact in preventing further episodes of heath anxiety in those who are currently well would also be of benefit. Given that the data shows that the impact of the MBCT programme increases over time and its effects are long lasting in people suffering from a current episode, this is likely to be a fruitful area of inquiry. Additionally, given the significant variability in response across participants (for some the improvement is minimal, for some it is very large, e.g. a change of 29 on the SHAI), future research could usefully look at who benefits most, and least, from such interventions and how the MBCT intervention might also be further tailored to the psychological processes evident in this population.
